# Evaluation of force systems generated by Memory Titanol^®^ springs with different preactivation bends

**DOI:** 10.1590/2177-6709.29.5.e242430.oar

**Published:** 2024-10-04

**Authors:** Henrique Barcelos BRANDÃO, Jonas BIANCHI, Lucas Arrais de CAMPOS, Alyssa Schiavon GANDINI, Luiz Gonzaga GANDINI

**Affiliations:** 1 São Paulo State University, School of Dentistry, Department of Morphology and Child Clinic - Orthodontics (Araraquara/SP, Brazil)

**Keywords:** Orthodontics, corrective, Biomechanical phenomena, Tooth movement techniques, Ortodontia corretiva, Fenômenos biomecânicos, Técnicas de movimentação dentária

## Abstract

**Objective:**

This study evaluated the force system generated by the Memory Titanol® spring (MTS) with different preactivation bends using an orthodontic force tester (OFT).

**Methods:**

Three preactivations were tested using a 0.017 × 0.022-in stainless steel (SS) wire and a 0.018 × 0.025-in NiTi segment, with an activation of 30º in the posterior segment (β), with 0º (Group 1 [G1]), 45º (Group 2 [G2]), and 60º (Group 3 [G3]) in the anterior segment (α).

**Results:**

The molars showed extrusion values of −1.33 N for G1 and −0.78 N for G2, and an intrusion value of 0.33 N for G3. The force in the premolars was intrusive with a variation of 1.34 N for G1 and 0.77 N for G2; and extrusive with a variation of −0.31 N for G3. Regarding the upright moment (Ty) of the molar, a distal moment was observed with values of 53.45 N.mm for G1 and 19.87 N.mm for G2, while G3 presented a mesial moment of −6.23 N.mm. G1, G2, and G3 all exhibited distal premolar moments (Ty) of 3.58, 2.45, and 0.68 N.mm, respectively.

**Conclusions:**

The tested preactivations exerted an extrusive force in G1 and G2 and an intrusive force in G3 during molar vertical movement. The premolar region in G1 and G2 showed intrusive force and distal moment.

## RESUMO

**Objetivo:**

Este estudo avaliou os sistemas de forças gerados pela Memory Titanol® Spring (MTS) com diferentes curvaturas de pré-ativação, utilizando um sensor de força ortodôntica (Orthodontic Force Tester, OFT).

**Métodos:**

Foram testadas três pré-ativações usando um fio de aço inoxidável 0,017” × 0,022” e um segmento de fio NiTi 0,018” × 0,025” com ativação de 30º no segmento posterior (β) e 0º (Grupo 1 [G1]), 45º (Grupo 2 [G2]) ou 60º (Grupo 3 [G3]) no segmento anterior (α).

**Resultados:**

Os molares apresentaram valores de extrusão de -1,33 N para G1 e -0,78 N para G2, e valor de intrusão de 0,33 N para G3. A força nos pré-molares foi intrusiva, com variação de 1,34 N para G1 e 0,77 N para G2; e extrusiva para G3, com variação de -0,31 N. Em relação ao momento vertical (Ty) do molar, observou-se momento distal com valores de 53,45 N.mm para G1 e 19,87 N.mm para G2, enquanto G3 apresentou momento mesial de -6,23 N.mm. G1, G2 e G3 exibiram momentos distais dos pré-molares (Ty) de 3,58, 2,45 e 0,68 N.mm, respectivamente.

**Conclusão:**

As pré-ativações testadas exerceram uma força extrusiva em G1 e G2 e uma força intrusiva em G3 durante o movimento vertical dos molares. A região dos pré-molares em G1 e G2 apresentou força intrusiva e momento distal.

## INTRODUCTION

The loss of the first permanent molar usually causes the second lower molars to tip mesially; the premolars, canines, and incisors to move distally; and a progressive vertical bone loss in this area.^1^ The solution is to upright the molar and, subsequently, conduct prosthetic rehabilitation or close this space with the natural dentition. The uprighting of the molar improves periodontal health and occlusion, allowing for the alignment of the perpendicular roots to the occlusal plane to resist the occlusal forces better.^2^

The literature describes many mechanical techniques for uprighting lower molars, including tip-back simple mechanic,^3^ cantilevers,^4^ double cantilevers,^5^ T-loop springs,^6^ vertical springs,^7^ and nickel-titanium (NiTi)-superelastic (SE)-stainless steel (SS) upright springs, commonly known as the Memory Titanol^®^ spring (MTS).^8^ Sander and Wichelhaus^9^ proposed three mechanisms: (1) Upright plus molar intrusion, (2) upright plus extrusion, and (3) upright with root movement. No published articles have shown that the force system generated by the Sander spring is compatible with the proposed results. Therefore, as indicated, the decision was to test and describe the mechanical effects generated by different preactivations of the MTS.

This study evaluated the force systems generated by the MTS on the segment of molars and premolars, with different preactivation protocols on the anterior segment (α), using load cells in an orthodontic force tester (OFT) to read the force systems generated on both sides of the system (α and β). Specifically, it examined an activation of 30º in the posterior segment (β), with 0° (Group 1 [G1]), 45° (Group 2 [G2]), and 60° (Group 3 [G3]) in the anterior segment (α).

## MATERIAL AND METHODS

An orthodontic cast from a patient with loss of the first lower left molar was selected. The inclination of the second lower left molar was measured with a template manufactured with a protactor^10^ relative to the occlusal plane.

**Figure 1:**
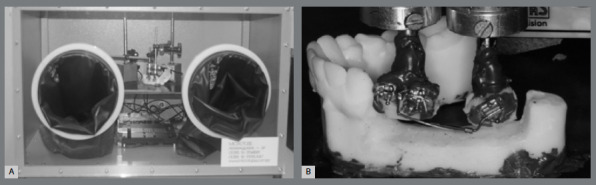
A) System inserted in a glove box-type acrylic case at a temperature of 37ºC. B) Memory Titanol^®^ spring (MTS) being measured by the OFT.

This cast was digitized with a 3Shape table scanner (Copenhagen, Denmark) and processed with the Meshmixer software (Autodesk, Inc., San Rafael, CA, USA). The first and second premolars and the second molar had their dental crowns cropped to be printed separately from the dental arch using a 3D printer (Form2; Formlabs, Somerville, MA, USA) for future fixation on the OFT^11^ (Advanced Research and Technology Institute, Inc., Indianapolis, IN, USA; Patent US 6.120.287, 2000; Fig. 1). A second full lower cast had the teeth on the left side impressed with heavy silicone (Zetalabor; Zhermack, Badia Polesine, Italy) to serve as a guide.

The separately printed teeth were fixed on the cast base with wax (ASFER Indústria Química Ltda., São Caetano do Sul, Brazil) using a silicon guide, which was removed after the fixation was complete. Then, the cast with the individually printed teeth was fixed to the base of the OFT^11^ with epoxy glue (JB Weld; Sulphur Springs, TX, USA). Roth Sprint brackets (0.022-in; Forestadent, Pforzheim, Germany) were bonded to the lower left premolars with the same epoxy glue, and a double Roth tube (0.022”) was bonded to the second lower left molar. A SS wire (0.019 × 0.022-in; Ormco; Glendora, CA, USA) was placed on the anterior segment, and a crossed tube (0.022-in; Forestadent, Pforzheim, Germany) was crimped between the premolars. The wire was tied to the bracket with elastic ligatures (GAC, Bohemia, NY, USA). Triad gel (Dentsply Sirona, Long Island City, NY, USA) was placed on the tips of the steel wire of the tip-back spring, to protect against lip injury. OFT load cells were fixed between the lower-left premolars, identified as T1, and the lower left molar, identified as T2 (Multi-Axis Force/Torque Nano17; ATI Industrial Automation, Apex, NC, USA), both with measured amplitudes of 0-2,000 g (0.0-19,613.3 N) for force and between 0-10,000 g.mm (0.0-98,006.5 N.mm) for moments. After the load cells were fixed, the wax was removed to allow the teeth to move, fixed on the extremities of the load cells.

The OFT sensors were adjusted to transfer the origin of the 3D measurements to the criss-cross tube between the lower-left premolars and the double tube on the lower-left molar, with the X-axis perpendicular, the Y-axis parallel, and the Z-axis vertical relative to the occlusal plane.

**Figure 2:**
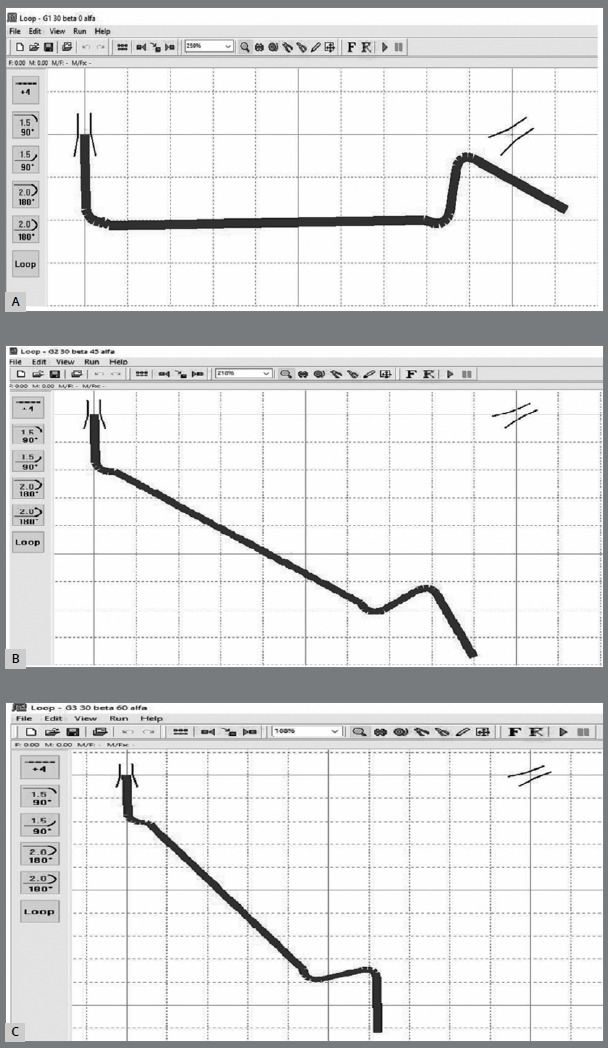
A) Preactivation template of Group 1. B) Preactivation template of Group 2. C) Preactivation template of Group 3.

The three examined groups had the same 30^o^ activation on the posterior segment (β), but different preactivation for the anterior segment (α): 0^o^ (G1), 45º (G2), and 60º (G3). The activation in the posterior segment did not include molar inclination, so the total of both would be 62°. Each group comprised five MTS, measuring 0.017 × 0.022-in on the SS segment and 0.018 × 0.025-in on the NiTi segment (Forestadent, Pforzheim, Germany). The template of the Sander spring, with each angulation on the SS segment, was drawn using the Loop software (dHAL Software, Athens, Greece; Fig 2).

Before each measurement, the OFT software and the load cells were reset to zero. The entire system was inserted into a 37ºC temperature-controlled acrylic case.^12^ The measurement of the forces for each sample was done twice. The force and moment values on the z-axis and y-axis (Fz and Ty) were tabulated on a spreadsheet compatible with Excel 2010 (Microsoft Office; Microsoft, Redmond, WA, USA). The data were analyzed using the SPSS software (version 20.0; IBM, Armonk, NY, USA). The reproducibility between the two measurements of the Fz and Ty forces in each sample was evaluated using the interclass correlation coefficient (ICC) and its 95% confidence interval (CI), considering the absolute accuracy and the variance analysis cast of two mixed factors for unique measurements.^13^ The accuracy between the measures was determined,^14^ showing an excellent value (Fz: ICC = 0.992 [95% CI = 0.983-0.996]; Ty = 0.990 (95% CI = 0.979-0,995). Subsequent analyses used the simple arithmetic average between the two measurements for each sample.

Fz and Ty were descriptively analyzed for each tooth in the group. The normality of the distribution was evaluated using the Shapiro-Wilk test. The homoscedasticity was assessed using Levene’s F test. When homoscedasticity was accepted, Fz and Ty were compared between the groups using a one-way analysis of variance (ANOVA) followed by Tukey’s *post-hoc* test. When homoscedasticity was rejected, they were compared using ANOVA with Welch’s correction followed by the Games-Howell *post-hoc* test.

## RESULTS

Table 1 shows the means, standard deviations, minimums, and maximums for each molar and premolar. The molars in G1 and G2 exhibited extrusion, with values of −1.33 and −0.78 N, respectively. In contrast, the molars in G3 exhibited intrusion, with a value of 0.33 N. In the anterior lower premolar region, the force was predominantly intrusive, ranging from 0.77 N for G2 to 1.34 N for G1, but tended to be extrusive in G3, with a value of −0.31N. The molars’ inclination moment (Ty) was distal in G1 and G2, with values of 53.45 and 19.87 N.mm, respectively, and mesial in G3, with a value of −6.23 N.mm. Premolars exhibited distal Ty moments in G1, G2, and G3.

**Table 1:** Descriptive statistics of Fz (N), vertical force, and descriptive statistics of Ty (N-mm), Moment in Sagittal Plane, according to tooth and group.

	Fz				Ty			
Group	Mean	SD	Min	Max	Mean	SD	Min	Max
Molar								
G1	-1.33	0.04	-1.38	-1.29	53.45	0.42	52.83	53.89
G2	-0.78	0.05	-0.86	-0.73	19.87	2.39	17.63	22.7
G3	0.33	0.02	-0.36	-0.3	-6.23	0.19	-6.5	-6.04
Premolar								
G1	1.34	0.03	1.31	1.4	3.58	0.16	3.35	3.72
G2	0.77	0.07	0.69	0.85	2.45	0.22	2.14	2.76
G3	-0.31	0.03	0.26	0.34	0.68	0.03	0.65	0.73

Table 2 shows the mean difference between the three examined groups. A difference can be observed for each tooth in at least one pair of groups.

**Table 2:** Mean comparison of forces and moments among the three groups.

Force		Sum of squares	Mean Square	F	p	η^2^p
Molar						
Fz	Between groups	2.547	1.274	928.510	<0.001	0.990
	Within groups	0.016	0.001			
	Total	2.564				
Ty	Between groups	8948.569	4474.285	37397.784*	<0.001	0.986
	Within groups	23.646	1.970			
	Total	8972.215				
Premolar						
Fz	Between groups	2.682	1.341	585.782	<0.001	0.990
	Within groups	0.027	0.002			
	Total	12.519				
Ty	Between groups	21.412	10.706	437.435	<0.001	0.986
	Within groups	0.294	0.024			
	Total	96.746				

*Welch correction.

Table 3 compares the forces and moments among the three examined groups. All groups exhibited distinct forces and moments.

**Table 3:** Mean and standard deviation of the variables Fz (N) and Ty (N-mm), according to tooth and group.

Group	Fz	Ty
Molar		
G1	-1.33 (0.04)^A^	53.45 (0.42)^C^
G2	-0.78 (0.05)^B^	19.87 (2.39)^B^
G3	0.33 (0.02)^C^	-6.23 (0.19)^A^
Premolar		
G1	1.34 (0.03)^C^	3.58 (0.16)^C^
G2	0.77 (0.07)^B^	2.45 (0.22)^B^
G3	-0.31 (0.03)^A^	0.68 (0.03)^A^

Superscript letters indicate the comparison among groups, considering the same tooth and force; different letters indicate statistical difference, Tukey or Games-Howell test, α=0.05.

## DISCUSSION

**Figure 3:**
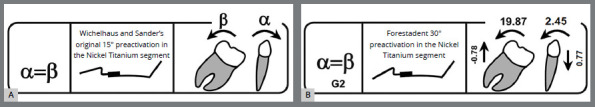
Figure adapted from the article by Wichelhaus and Sander (1995). A) Sander’s proposal to activation on G2. B) G2 in the present study.

This study evaluated the force systems generated by the MTS and compared them with the different preactivation bends in the SS wire segment proposed by Wichelhaus and Sander.^8^ The focus was on the sagittal plane, the vertical forces, and moments on this specific plane. The values in Table 1 suggest the same direction of movement for G1 and G2, but a different direction for G3. The original hypothesis could be confirmed, potentially leading to increased inclination of the anterior segment and possibly neutralizing extrusive force in the posterior segment. While G1 and G2 showed posterior extrusive and anterior intrusive forces, G3 showed the opposite. Wichelhaus and Sander^8^ proposed molar upright and premolar mesial moment with α = β activation, but this remains unverified, considering this paper did not test this scenario. When 30° posteriorly and 45° anteriorly were tested (G2), the main difference was in the resulting force system; instead of having just moments on the anterior and posterior segments, the class VI geometry of Burstone and Koenig,^15^ this study observed class III geometry, which includes vertical forces and moments in the same direction (Fig 3).

**Figure 4:**
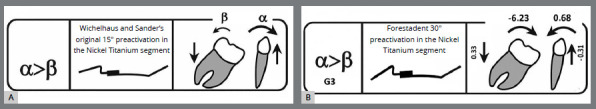
Figure adapted from the article by Wichelhaus and Sander (1995). A) Sander’s proposal to activate on G3. B) G3 in the present study.

**Figure 5:**
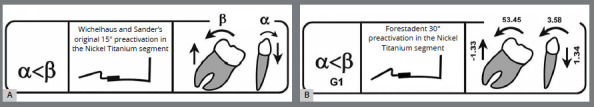
Figure adapted from the article by Wichelhaus and Sander (1995). A) Sander’s proposal to activate on G1. B) G1 in the present study.

When the activation of α was greater than that of β, the molar underwent uprighting with intrusion, and the premolars underwent extrusion and a distal moment (Fig 4). Conversely, when the activation of α was less than that of β, the molar underwent uprighting with extrusion, and the premolars underwent intrusion and a distal moment (Fig 5). The original hypothesis suggested that the anterior preactivation moment would produce an extrusive force in the molar area. However, this study found that the generated force system did not align with Burstone’s class VI geometry but with the “V” bend shifted posteriorly. Consequently, the extrusive force remains in the molar for G1 and G2.

Compared to this study, the behavior described by Wichelhaus and Sander^8^ related to the anterior segment activated less than the posterior, which is again incongruent. This activation generated a force system with class III geometry in this study. Wichelhaus and Sander^8^ described anterior rotation on the premolar, while this study found posterior rotation on the anterior area. The Wichelhaus and Sander^8^ system generated a class V geometry, but this study observed class III geometry.

According to Wichelhaus and Sander^8^, the uprighting moment is small in less inclined molars. In order to avoid further activation, the tip-back angulation must be increased to 30°, generating a more significant moment in the molar and reducing the need for spring reactivation.^8^ An SS wire anchorage provides stabilization due to its high rigidity alloy, allowing the NiTi alloy to function flexibly, imparting moment to the molar. This study showed that the SS wire does not behave desirably, probably due to combining a rigid wire with a flexible one, which would consequently have a class III geometry.^15^ The theoretical idea of a rigid wire in the stability area, and a flexible wire in the region with more movement did not produce the expected results in this study.^16^

In photoelasticity studies, Pinheiro^17^ demonstrated that the 0º and 45º activations on the anterior segment proposed by Sanders created stress areas on the molar side, compared to a simple cantilever, meaning all devices showed a distal upright moment. That study did not evaluate the vertical forces. In contrast, in the reactive anterior region of the premolars, the Sander spring with 0º of activation in the anterior segment had a similar behavior to the simple cantilever. This data is incompatible with this study, which can be explained by the fact that the study by Pinheiro^17^ was qualitative, while this study was quantitative and used a more sensible system to measure forces and moments.

The moment’s intensity is ideal for the upright molars, since it was 10-15 N.mm.^4^ With that in mind, among all groups tested in this study, G2 behaved best (Ty from the molar = 17.63 N.mm). G1 had excessive moments of around 50 N.mm, and G3 had moments in the opposite direction to that desired.

The vertical force in the molar area decreased as the preactivation was increased in the anterior segment of the SS, with G3 having the lower molar intrusion force and, consequently, extrusion in the anterior segment. However, the upright moment was unfavorable. G2 had the best biomechanical cost-benefit.

Shibasaki and Martins^18^ compared different tube heights on inclined molars using continuous NiTi wires (0.016 × 0.022-in) and an OFT for measurements. They concluded that a force system always generates posterior extrusive and intrusive anterior forces. These data are compatible with this study, including the intensity of the forces and moments and the Burstone class III geometry pattern. The vertical forces varied between −0.75 and −1.31 N and the moments, between 11.84 and 13.98 N.mm on the molar. Therefore, the choice between continuous wires and the MTS, G2, would be more closely related to a personal preference than the mechanical factors on the force systems. Shibasaki and Martins^18^ also suggested using a tube placed more occlusal on the molar, which, from a clinical standpoint, may be impractical due to contact with the antagonist. If the orthodontist still chooses to raise the bite, the extrusive forces manifest with greater ease, due to the absence of antagonist occlusal forces.

Finally, from a clinical practice standpoint, the MTS performed best in G2, since it was similar to a simple cantilever made of titanium and molybdenum alloy (0.017 × 0.025-in), exhibiting vertical moment and extrusion force. This system generated a Class III geometry, which also presents a moment in the anterior segment, but a relatively weak extrusive force, averaging 0.78 N, that can be neutralized by occlusal forces if the patient has a normal or hypodivergent facial pattern.^19^

However, the force systems generated by the cantilevers and the MTS would differ in the anterior region. The former would generate an intrusive force and possibly some moment force, depending on the line of action, related to the center of resistance of the anchorage segment. The latter generates distal binary moments because it is inserted in a criss-cross tube.

## CONCLUSIONS

1. G1 and G2 exhibited extrusive forces on the molar and intrusive forces on the premolars, while G3 exhibited intrusive forces on the molar.
2. The distal moment generated in G1 had an high intensity.
3. G3 resulted in an unfavorable molar moment in the mesial direction.
4. The preactivation in G2 was the most favorable regarding direction and intensity.
5. Regarding the anchorage on the anterior region, all groups showed moments in a distal direction.
